# Tannin Content in *Vitis* Species Red Wines Quantified Using Three Analytical Methods

**DOI:** 10.3390/molecules26164923

**Published:** 2021-08-14

**Authors:** Aude A. Watrelot

**Affiliations:** Department of Food Science and Human Nutrition, Iowa State University, 536 Farm House Lane, Ames, IA 50011-1054, USA; watrelot@iastate.edu; Tel.: +1-515-294-0343

**Keywords:** proanthocyanidins, interspecific hybrid grapes, methylcellulose precipitation, protein precipitation, HPLC-PLRP

## Abstract

Tannin content in red wines is positively correlated with astringency perception and wine grade; however, tannin quantification is one of the main challenges. In this study, tannin content was quantified using three analytical methods in commercial red wines from *Vitis vinifera* and interspecific cold-hardy hybrids including Marquette, Frontenac, and Petite pearl cultivars. Protein (PP) and methylcellulose precipitation (MCP) methods were compared to a HPLC-DAD method, which is based on the interaction between tannins and a hydrophobic surface (RPC). Frontenac wines were the poorest in tannins and Cabernet sauvignon wines were the richest regardless of the method used. In cold-hardy red wines, the tannin content was higher in Marquette with high alcohol content, which suggested that the tannins were extracted from seeds rather than skins. The high limit of quantification of the PP method and the presence of anthocyanin di-glucosides in cold-hardy wines were parameters suggesting that protein and methylcellulose precipitation methods were neither suitable nor reliable for the quantification of tannins in cold-hardy red wines. The tannin content quantified by RPC was positively correlated to tannin quantified by MCP, suggesting that the RPC method would be relevant for the quantification of tannins in red wines.

## 1. Introduction

In red wine, tannins are considered to be of fundamental importance to quality, as they impart astringency [1]. Based upon information from wine reviews, astringency is generally described using descriptive terms having quality implications (silky, grippy, unripe, coarse, etc.) [2,3]. Despite the prevalence of these terms in the wine producing and consuming population, it remains a challenge to manage these terms in the vineyard and winery. Much of the challenge can be related to insufficient analytical methodology for measuring astringency “qualities” in wine. In finished red wines, the balanced level of astringency is a very important characteristic for high quality. Red wine that is not astringent enough is commonly perceived to be “flat” or insipid and thus uninteresting for consumers. Meanwhile, red wines that are too astringent lead to unbalanced wine that detracts from other sensory characteristics, which is unfavorable. Astringency is generally considered to be a tactile sensation caused by the interactions between wine tannins and salivary proteins, followed by the precipitation of complexes and a loss of in-mouth lubrication [4]. The astringency/drying perception of red wine is strongly and positively correlated with the tannin concentration [5,6]. Condensed tannins are oligomer and polymer of flavan-3-ols, which are extracted from grape skins and seeds during alcoholic fermentation and extended maceration in red wine making [7]. Condensed tannins are characterized by the type of constitutive subunits linked mainly through C4-C8 linkages, and the number of these subunits provides an indication of the mean degree of polymerization [8,9]. As previously described, the size of condensed tannins is positively related to their ability to interact with proteins and polysaccharides. The larger the tannins, the stronger the binding affinities [10,11,12]. The chemical methods commonly used in the wine industry to measure astringency are based on tannin–protein or tannin–polysaccharide interaction and the precipitation of complexes, which positively correlates with the astringency perception. Mercurio and Smith (2008) [13] have previously shown that the astringency sensation of red wines was positively correlated with the tannin concentration determined by protein and methylcellulose precipitation (MCP). However, the tannin content determined by protein precipitation and methylcellulose precipitation methods is not consistent across the research [14], and it seems to be highly dependent on the method applied, the structure of the tannins, and the presence of other matrix compounds [15]. Those methods have been mainly applied to *Vitis vinifera* grapes and wines, but there is a lack of studies on the characterization of tannin content in interspecific cold-hardy grapes and wines [16,17,18,19,20]. The wines produced from cold-hardy grapes tend to be high in acidity, low in astringency, and rich in anthocyanin mono- and di-glucosides [21], which might interfere in the analysis of condensed tannins by the precipitation methods. An alternative method by high-performance liquid chromatography using a hydrophobic surface under a gradient of solvents (RPC) has been previously developed to characterize the tannin content and activity, but no correlation between the precipitation methods and this reversed-phase chromatography method in different red wines has been reported before [4,7,22,23]. The latter method is based on the ability of tannins to bind to a hydrophobic surface, but it does not discriminate depending on tannin structure or tannins already associated to macromolecules.

The aim of this study was to quantify tannins in red wines of the *Vitis* species and to compare the three analytical methods including protein, methylcellulose precipitation, and reversed-phase chromatography for this quantification.

## 2. Results

*Vitis vinifera* cultivars used in this study were Cabernet sauvignon and Pinot noir from California. Interspecific cold-hardy cultivars including Petite pearl, Frontenac, and Marquette from Montana and Iowa were used (Table 1). The vintages of Marquette wines varied from 2014 to 2019, and some Frontenac wines were 2015, 2017, and 2018. The vintage was not known for all of the wines. The pH of the wines varied from 3.35 in 2015 Frontenac (F2) to 3.99 in 2018 Frontenac (F4). The alcohol content was the highest in Marquette M4 at 15.68% (*v*/*v*) and the lowest in Frontenac F6 at 10.07% (*v*/*v*). The pH and alcohol content of *Vitis vinifera* wines showed less variation than in cold-hardy interspecific hybrid wines (pH varied from 3.60 to 3.78 and alcohol content varied from 13.54 to 13.68 in *V. vinifera* cv. Pinot noir and Cabernet sauvignon).

### 2.1. Phenolic Compounds and Tannin Content Per Variety

A total iron-reactive phenolic compounds method was used in this study to evaluate the content of phenolics reacting with ferric chloride over the Folin–Ciocalteu method. This latter method is based on the assumption that the main antioxidants in plants are phenolic compounds. However, Folin–Ciocalteu reagent can react with different antioxidants such as amino acids, carbohydrates, vitamin C, etc. In interspecific cold-hardy wines, the total phenolics content was not significantly different between cultivars and was varying between 911 and 1098 mg/L of (+)-catechin equivalent (Table 2). In interspecific cold-hardy cultivars, the coefficient of variation of phenolic content was high, and it varied between 42.7% in Petite pearl, 22.4% in Frontenac, and 58.3% in Marquette wines (not shown). The high coefficient of variation in those cultivars may be attributed to the diversity of vintage and location. The tannin content in red wines was dependent on the cultivars. As previously observed [24,25,26], *Vitis vinifera* wines contained the highest mean tannin content, and Cabernet sauvignon wines showed the highest content from 557 to 2045 mg/L depending on the method (PP versus MCP) (Table 2). Within the cold-hardy interspecific cultivars, Frontenac wines contained the lowest average tannin content varying between 48 and 412 mg/L depending on the method.

**Figure 1:**
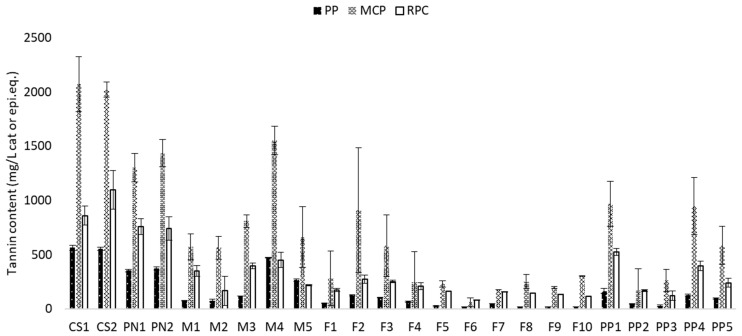
Tannin content in red wines of different varieties using the three analytical methods.

The tannin content in wines from interspecific species was highly variable compared to *Vitis vinifera* wines, which was likely due to the low number of Cabernet sauvignon and Pinot noir wines (Figure 1). Within cv. Marquette, the tannin content was the highest in M4 at 470 mg/L with the PP method and was the lowest in M1 and M2 with the same method (75 mg/L). Within cv. Frontenac, the tannin content was the lowest in F6 at 17 mg/L with the PP method (below the limit of quantification) and the highest in F2 and F3 at 123 mg/L with the same method (Figure 1). For the Petite pearl cultivar, the tannin content was the lowest in PP2 and PP3.

### 2.2. Tannin Content Measured by Three Methods

**Figure 2:**
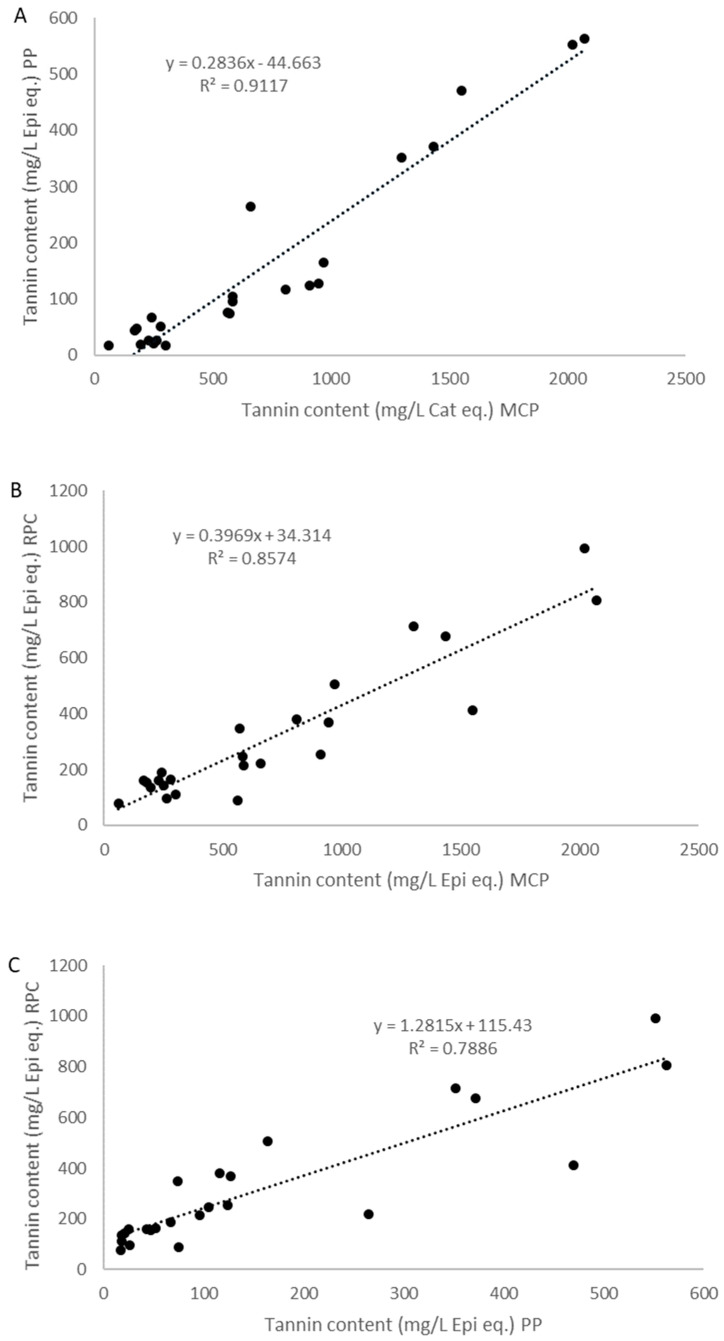
Linear regression analysis of tannin content in red wines of Cabernet sauvignon, Pinot noir, Petite pearl, Marquette, and Frontenac varieties determined using methylcellulose precipitation (MCP), protein precipitation (PP) (**A**), and reversed-phase chromatography (RPC) methods (**B**,**C**).

As shown in Table 2, the concentration of tannins varied depending on the method used. The content was 3.7 times higher in *Vitis vinifera* wines when measured by the methylcellulose precipitation compared to the protein precipitation method [13,27]. The strongest correlation of wine tannin content was observed between the two precipitation methods, as previously shown [13,27]. The linear regression of the tannin content in the twenty-four wines between PP and MCP methods showed a R-squared value of 0.91 (Figure 2A), which decreased to 0.74 when *Vitis vinifera* wines were excluded (not shown). This correlation was in agreement with published correlations between PP and MCP methods on *Vitis vinifera* red wines, as Mercurio and Smith [13] observed with a R-squared value of 0.79 in 44 red wines. However, the correlation between PP and MCP of *Vitis* species wines was higher than previously observed by Cáceres-Mella [27] on 20 Cabernet sauvignon red wines (R = 0.58). The linear regression of the tannin content in all wines between RPC and MCP methods showed a strong correlation with a R-squared value of 0.87 (Figure 2B), which decreased to 0.66 when *Vitis vinifera* wines were excluded (not shown). The linear regression of the tannin content in all wines between RPC and PP methods showed a strong correlation with a R-squared value of 0.78 (Figure 2C), which decreased to 0.39 when *Vitis vinifera* wines were excluded (not shown). As previously observed [13,15], the slope of the linear regression between the precipitation methods showed an almost 0.3-fold difference in tannin content, and the intercept indicated that the MCP method led to the precipitation of more tannin material than the PP method (Figure 2A). Similarly, the slope of the linear regressions between RPC and the precipitation methods indicated an almost 0.4-fold and 1.3-fold difference in tannin content between RPC vs. MCP and RPC vs. PP, respectively (Figure 2B,C).

## 3. Discussion

The concentration of tannin was evaluated using three analytical methods in twenty-four red wines of five varieties (Table 1), including *Vitis vinifera* and interspecific cold-hardy hybrids. Cabernet sauvignon and Pinot noir wines were the richest in phenolic compounds, tannins, and cv. Frontenac wines showed the lowest tannin content. About one-third of phenolic compounds in all red wines was tannin. The tannin content in Cabernet sauvignon and Pinot noir measured by precipitation methods was in the same range, i.e., ≈400 mg/L by protein precipitation, as previously published [13,24,25,26,27]. Interspecific cold-hardy red wines such as Maréchal Foch, Léon Millot, Marquette, Frontenac, etc. are known for their low percentage of tannin extractability and therefore low tannin content [19,25]. Little research has been conducted on the analysis of tannin content in cold-hardy grapes, especially on Marquette, Frontenac, and Petite pearl red wines. In Marquette wines, the average tannin content was higher than in previous published studies [28,29], which was most likely because of the analytical method used (MCP versus HPLC-DAD after acid-catalysis), the lack of repeatability of the precipitation methods, and the large variability within the same cultivar. Similar to the MCP method, the PP method has been previously shown as a not reliable method for French-American and neo-American hybrid red wines [25]. In Frontenac wines, the average tannin content was below the limit of quantification of the protein precipitation method as previously observed, and it decreased after the end of alcoholic fermentation, which was possibly due to interactions between proteins and tannins [19]. In Petite pearl wines, the average tannin content was also found to be lower than *Vitis vinifera* wines, and, to the best of our knowledge, this was the first data about tannin content in Petite pearl red wines. The concentration of phenolic compounds and tannins is influenced by many factors including viticultural and winemaking practices as well as the cultivars and wine matrix. Within the cultivars, the tannin content largely varied as observed in Marquette wines, in which M4 showed the highest tannin content, and in Frontenac wines, where F6 showed the lowest tannin content. M4 was a wine from Montana of 2017, which was high in alcohol compared to other Marquette of the same vintage from other location. F6 was a wine from Iowa with an unknown vintage but with a very low alcohol content (≈10% *v*/*v*). During the wine-making process, tannins are extracted slowly from grapes, first from skins and then from seeds with the increase of ethanol content produced during alcoholic fermentation [30]. As previously shown by Rice et al., tannins are mainly found in seeds of Marquette and Frontenac grapes rather than in skins, i.e., 0.26 and 0.54 mg/berry expressed as (+)-catechin equivalent in Frontenac and Marquette seeds, respectively, versus 0.05 and 0.14 mg/berry expressed as (+)-catechin equivalent in Frontenac and Marquette skins, respectively [17]. This suggests that the high tannin content in M4 and low tannin content in F6 was related to the level of alcohol in the finished wine, which facilitated the extraction of tannins from seeds.

Tannin content is highly variable depending on the method used and is often an issue when trying to quantify those compounds. Precipitation methods using either a protein, such as the bovine serum albumin, or a polysaccharide, such as methylcellulose, are well developed in laboratories and wineries. Those two methods are based on the ability of tannins to bind to proteins and polysaccharides to form a complex and precipitate. They have pros such as ease of practice and strong, positive correlation with perceived red wine astringency [13,15], but they also have cons including lack of reliability in non-*Vitis vinifera* wines due to the high limit of quantification [25]. Moreover, an almost three-fold difference is commonly observed between PP and MCP methods where MCP has been shown to remove more tannin material than PP in *Vitis vinifera* wines [13]. The difference between PP and MCP methods for tannin content in interspecific hybrid wines was similar to that of *Vitis* *vinifera* wines with an almost three-fold difference. The PP method seemed to evaluate tannins or polymeric pigments that are larger than dimer and trimer, while the MCP method removed all of the tannins, from dimer to polymer [31]. Another difference is that the PP method determines the concentration of tannins after reacting with iron chloride, whereas the MCP value is based on the direct absorbance values at 280 nm. The other method used in this study, RPC, was also based on the absorbance values at 280 nm of tannins that are able to interact with a hydrophobic surface, the polystyrene divinylbenzene column by HPLC-DAD. As expected, the correlation of tannin content quantified by MCP and RPC was strong compared to PP and RPC, which was most likely because the detection was carried out at the same wavelength. The MCP method has been shown to quantify total tannin and pigmented tannins, as those compounds are able to interact and precipitate with methylcellulose [32]. However, the method of quantification is using the comparison of the absorbance values at 280 nm of the supernatants before and after precipitation with methylcellulose. Anthocyanins show a maximum of absorbance at about 520 nm and also absorb at 280 nm. But, they do not precipitate with methylcellulose, leading to high absorbance values at 280 nm of the supernatants. In cold-hardy red wines, the type of anthocyanin and content of anthocyanins, di-glucoside, and mono-glucoside is much higher than in *Vitis vinifera* wines, e.g., Frontenac wines showed between 2 and 6 g/L of anthocyanins di-glucoside, while *Vitis vinifera* showed much lower content of anthocyanins mono-glucoside [33]. Those differences in anthocyanin structure lead to various color intensities and less pigmented tannins formed, but this seems to be a source of variation when quantifying tannins using the methylcellulose precipitation method and the absorbance reading at 280 nm. In most cold-hardy red wines, the absorbance values at 280 nm before and after methylcellulose precipitation shows a saturation of absorbance (above 2 absorbance units), and dilutions are required. However, in cold-hardy wines, the tannin content was very low, and a dilution step would lead to high variation and low repeatability. Further work will be investigating the effect of the structure and content of anthocyanins and pigmented tannins on the quantification of tannin content using those analytical methods. Therefore, our results, in agreement with the previously published HPLC-FLD method [19], suggested that the RPC method is a suitable and reliable method for the quantification of tannins in cold-hardy wines rich in anthocyanins mono- and di-glucoside.

## 4. Materials and Methods

### 4.1. Chemicals and Wine Samples

Hydrochloric acid, sodium hydroxide, glacial acetic acid, ferric chloride hexahydrate, *ortho*-phosphoric acid, acetonitrile, sulfuric acid, and 0.1 N sodium hydroxide were purchased from Fisher Scientific (Santa Clara, CA, USA). Sodium chloride, methylcellulose, bovine serum albumin (BSA), (−)-epicatechin, and (+)-catechin were purchased from Sigma-Aldrich (St. Louis, MO, USA). Potassium metabisulfite, sodium dodecyl sulfate, and ammonium dihydrogen phosphate were purchased from Acros Organics (Geel, Belgium). Triethanolamine was purchased from Aqua Solutions, Inc. (Deer Park, TX, USA).

Twenty-four commercial wines from *Vitis vinifera* and interspecific cold-hardy hybrids including two ‘Cabernet sauvignon’, two ‘Pinot noir’, five ‘Marquette’, ten ‘Frontenac’, and five ‘Petite pearl’ cultivars were purchased in a liquor store, and they were provided by wineries from Iowa and Montana. Table 1 is the list of commercial red wines including vintage, variety, and origin.

### 4.2. Wine Chemical Analyses

The pH of wines was measured with a digital pH meter (ThermoScientific^®^, model Orion Star A211 (Waltham, MA, USA). The alcohol content in red wines was analyzed using an alcohol and extract meter, Alex 500 (Anton Paar, Vernon Hills, IL, USA). The alcohol and extract meter is based on the near-infrared technique, and the alcohol content was evaluated from 20 mL of red wines at room temperature.

### 4.3. Wine Total Iron-Reactive Phenolic Compounds Content

The total iron-reactive phenolics content in red wines was quantified following a previously published manuscript [34]. Briefly, 75 µL of centrifuged red wine was added in a 1 mL cuvette, and 800 µL of buffer containing sodium dodecylsulfate (50 g/L) and triethanolamine (50 mL/L) with a pH of 9.4 was added and incubated after vortexing for 10 min at room temperature. The absorbance at 510 nm was recorded using a UV-Visible spectrophotometer (Thermo Scientific™ GENESYS™ 140/150 UV-Vis spectrophotometer, Fisher scientific, Madison, WI, USA). Then, 125 µL of acidified ferric chloride (2.7 g/L) solution with hydrochloric acid (800 µL/L) was added, vortexed, and incubated for 10 min at room temperature. The absorbance at 510 nm was recorded, and the total iron-reactive phenolic compound content in red wines was determined as the difference of the final and initial absorbance values, and it was expressed as (+)-catechin equivalent using a (+)-catechin calibration curve. The analyses were performed in triplicate for each wine.

### 4.4. Wine Tannin Content

Total wine tannin concentrations in red wines were determined using three previously published methods.

Methylcellulose precipitation assay (MCP) [32]. Briefly, a methylcellulose solution of 0.04% and a saturated ammonium sulfate solution were prepared. First, 25 µL of centrifuged wine supernatants were placed in two centrifuge tubes. In the treatment tubes, 300 µL of methylcellulose solution was added, followed by 200 µL of ammonium sulfate solution and 400 µL of water. In the control tubes, 300 µL of water was added, which was followed by 200 µL of ammonium sulfate solution and 400 µL of water. After gentle shaking and incubation for 10 min at room temperature, the tubes were centrifuged at 16,200× *g* for 5 min using an Accuspin Micro17 centrifuge (Fisher Scientific). The supernatants were transferred in a UV-Visible cuvette, and the absorbance was recorded at 280 nm. The tannin content was determined as the difference between the control and treatment based on the dilution factor. The content was expressed as (−)-epicatechin equivalent using an (−)-epicatechin calibration curve. The analyses were performed in triplicate for each wine.

Protein precipitation assay (PP) [24]. All the buffers needed for this analysis were prepared according to the previously published method from Harbertson et al. [24]. Bovine serum albumin was the protein used for the method at a concentration of 1g/L. Depending on the iron-reactive phenolic compounds content, the volume of centrifuged wines varied between 166.6 and 500 µL. After the addition of 1 mL of protein solution, the samples were incubated for 15 min prior to centrifuging at 16,200× *g*. The supernatants were discarded, and the pellets were rinsed and re-solubilized in the buffer containing sodium dodecylsulfate (50 g/L) and triethanolamine (50 mL/L) at a pH of 9.4. After vortexing and then incubation for 20 min, the absorbance at 510 nm was recorded using a UV-visible spectrophotometer. Then, 125 µL of acidified ferric chloride (2.7 g/L) solution with hydrochloric acid (800 µL/L) was added, vortexed, and incubated for 10 min at room temperature. The absorbance at 510 nm was recorded, and the total tannin content in red wines was determined as the difference of the final and initial absorbance measurements. The contents were expressed as (+)-catechin equivalent using a (+)-catechin calibration curve. The analyses were performed in triplicate for each wine.

**Figure 3:**
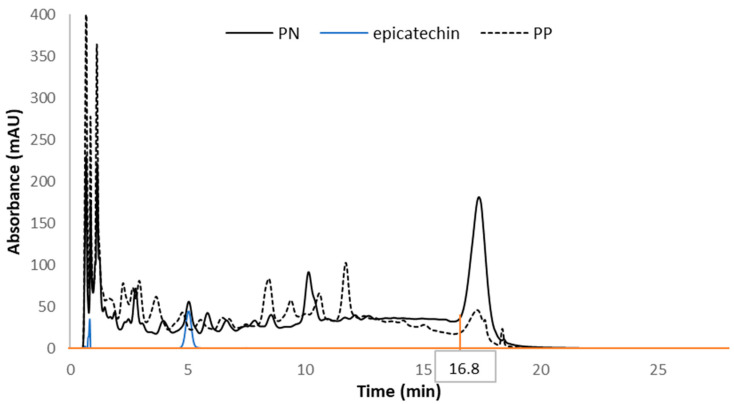
RPC chromatograms of Pinot noir (PN), Petite pearl (PP) wines and (−)-epicatechin after subtraction of water blank. The orange lines correspond to the baseline drawn at 0 mAU across the chromatogram and to the split area at 16.8 min when the gradient changed.

Reversed-phase chromatography method (RPC) [22]. The tannin content in red wines was quantified using a polystyrene divinylbenzene reversed-phase column (PLRP-S, 2.1 × 50 mm, 100 Å, 3 μm, Agilent Technologies) protected with a guard column (PRP-1, 3 × 8 mm, Hamilton Co., Reno, NV, USA) and analyzed by high-performance liquid chromatography with a diode array detector (HPLC-DAD, 1260 Infinity II, Agilent Technologies, Santa Clara, CA, USA). This system consisted of a model G7111 quaternary pump and degasser, a G7129 autosampler, a G7116 column oven, and a G7115 diode array detector. The data were processed using OpenLab ChemStation 3D UV software. The method has been modified from the previously published method as follows. The mobile phases consisted of 1.5% (*w*/*w*) 85% ortho-phosphoric acid in water (mobile phase A) and 20% (*v*/*v*) mobile phase A in acetonitrile (mobile phase B) with a flow rate of 0.30 mL/min. The linear gradient was as follows: time in min (% B), 0 (14%), 12.6 (34%), 12.6−13.3 (34%), 15.1 (70%), 15.1−16.8 (70%), 19.6 (14%), and 19.6−28.0 (14%). The oven was set up at 30 °C, and centrifuged red wine supernatants and water used as a blank were directly injected (5 µL). Following baseline subtraction of the water blank, chromatograms at 280 nm were integrated by establishing a baseline at 0 mAU across the entire chromatogram (Figure 3). Then, the peak area was split at 16.8 min, as this is the end of 70% of mobile phase B, as previously described by Barak et al. [22]. The peak area after 16.8 min elution was considered for the calculation of tannin content as it was attributed to polymeric material by Peng et al. [35], which was reported as (−)-epicatechin equivalent using an (−)-epicatechin calibration curve. The limits of detection (LOD) and quantification (LOQ) were calculated from the (−)-epicatechin calibration curve as 3.3 or 10 times the standard deviation of the response divided by the slope, respectively. The LOD was 40.7 mg/L and the LOQ was 123.5 mg/L as (−)-epicatechin equivalent. The analyses were performed in triplicate for each wine.

### 4.5. Statistical Analysis

Phenolic compounds and tannins in red wine data were analyzed with the XL Stat software (version 2021.2.1) using analysis of variance (ANOVA) and Tukey HSD analysis of the differences between the categories with a confidence interval of 95%. The results were expressed as means and standard deviation of triplicate analysis for each commercial wine. Linear regressions between the analytical methods used for tannin content quantification were carried out with XL Stat software.

## 5. Conclusions

Red wines produced from cold-hardy grape cultivars tend to show very low tannin content, with Frontenac wines being the lowest. Alcohol produced during fermentation enhances the extraction of tannins from grape seeds, and our results suggest that tannins present in cold-hardy red wines are mainly from seeds. Further work will be carried out on the chemical characterization of the structure of tannins from cold-hardy red wines. The common methods used to determine tannin content in red wines have been shown to be less relevant for cold-hardy cultivars than for *Vitis vinifera,* as a result of low tannin content, a high limit of quantification, and the presence of a high concentration of anthocyanins mono-and di-glucoside. The tannin content in commercial red wines quantified by the RPC method was 0.4-fold lower than by MCP and was 1.3-fold higher than by the PP method. A strong positive correlation was observed between PP and MCP methods, but the tannin content was 3-fold higher by MCP than by the PP method. In this study, the HPLC method (RPC) based on the interaction of tannins with a hydrophobic surface provided reliable and viable tannin content in all red wine cultivars. In contrast, the methylcellulose precipitation (MCP) method does not seem to be highly repeatable or accurate in cold-hardy red wines rich in anthocyanin di-glucoside. Further work will be carried out on the interference of anthocyanin di-glucoside in the three analytical methods used.

## Figures and Tables

**Table 1:** List of finished wines used in the study, with the vintage, variety, and origin. NA, not available.

Name	Vintage	Variety	Origin	pH	Alcohol (% *v*/*v*)
CS1	NA	Cabernet sauvignon	California	3.78	13.68
CS2	NA	Cabernet sauvignon	California	3.73	13.6
PN1	NA	Pinot noir	California	3.76	13.54
PN2	2017	Pinot noir	California	3.6	13.58
M1	2014	Marquette	Iowa	3.83	12.55
M2	2016	Marquette	Iowa	3.6	13.12
M3	2017	Marquette	Iowa	3.55	13.34
M4	2017	Marquette	Montana	3.75	15.68
M5	2019	Marquette	Montana	3.54	11.5
F1	2017	Frontenac	Iowa	3.67	12.14
F2	2018	Frontenac	Iowa	3.35	12.61
F3	2017	Frontenac	Iowa	3.36	14.51
F4	2018	Frontenac	Iowa	3.99	11.82
F5	2015	Frontenac	Iowa	3.5	12.52
F6	NA	Frontenac	Iowa	3.44	10.07
F7	NA	Frontenac	Iowa	3.37	12.48
F8	NA	Frontenac	Iowa	3.55	12.79
F9	NA	Frontenac	Iowa	3.36	12.35
F10	NA	Frontenac	Iowa	3.8	11.22
PP1	NA	Petite pearl	Iowa	3.59	13.7
PP2	NA	Petite pearl	Iowa	3.55	12.02
PP3	NA	Petite pearl	Iowa	3.53	12.34
PP4	NA	Petite pearl	Iowa	3.56	11.73
PP5	NA	Petite pearl	Iowa	3.69	11.7

**Table 2:** Total iron-reactive phenolic compounds content in finished red wines and tannin content in red wines determined by the three analytical methods.

Variety	Phenolics Content (mg/L cat. eq.)	Tannin Content (mg/L cat. eq.) (PP)	Tannin Content (mg/L epi. eq.) (MCP)	Tannin Content (mg/L epi. eq.) (RPC)
Cabernet sauvignon	1540 ± 91 ^a^	557 ± 18 ^a^	2045 ± 169 ^a^	978 ± 181 ^a^
Pinot noir	1234 ± 93 ^b^	362 ± 16 ^b^	1367 ± 137 ^b^	748 ± 82 ^b^
Petite pearl	930 ± 397 ^ab^	110 ± 51 ^c^	651 ± 379 ^cd^	289 ± 158 ^c^
Frontenac	911 ± 204 ^ab^	48 ± 38 ^d^	412 ± 392 ^d^	168 ± 60 ^d^
Marquette	1098 ± 640 ^ab^	200 ± 158 ^bc^	831 ± 406 ^c^	310 ± 134 ^c^

Cat, (+)-catechin; epi, (−)-epicatechin. Values not connected by the same superscript letter are significantly different (*p*-value < 0.05) among method.

## Data Availability

Not applicable.

## Sample Availability

Not available.
